# Newly Diagnosed Sjogren Syndrome in an Elderly Patient on Dialysis: A Case Report

**DOI:** 10.7759/cureus.36430

**Published:** 2023-03-20

**Authors:** Masatoshi Inoue, Momoko Sasamoto

**Affiliations:** 1 Nephrology, Tsushima City Hospital, Aichi, JPN

**Keywords:** glucocorticoid therapy, systemic autoimmune disease, elderly people, maintenance hemodialysis, primary sjogren’s syndrome

## Abstract

Sjogren syndrome (SS) is a chronic, systemic autoimmune disease that primarily affects the exocrine glands, causing dry eyes and mouth, but also presents with a variety of other symptoms. SS is a common connective tissue disease but it can be difficult to diagnose due to the non-specific symptoms and lack of diagnostic markers in many cases. This report describes a case of an elderly patient on dialysis with newly diagnosed SS. The patient had been unable to eat a normal diet for a year, but treatment had not been initiated, presumably because of his age and the fact that he was on dialysis. The patient's symptoms improved with the administration of glucocorticoids. This is a very educational case for physicians to recognize undiagnosed SS patients presenting with non-specific symptoms.

## Introduction

Sjogren syndrome (SS) is a systemic autoimmune disease that primarily affects the exocrine glands, causing dry eyes and mouth. However, patients with SS may require treatment with systemic immunosuppressive agents to manage a variety of extraglandular manifestations that cause symptoms such as severe fatigue, diffuse pain, fever, and dyspnea. Thus, the diagnosis of SS in the elderly is not easy, and the exclusion of other diseases presenting similar symptoms must be considered [1]. The most frequent form of nephropathy in SS is tubulointerstitial nephritis, which is a risk of chronic kidney disease [2]. Therefore, maintenance dialysis therapy could be initiated due to SS, but there are few reports of newly diagnosed SS in dialysis patients [3-4]. This report gives the details of a case of an elderly dialysis patient diagnosed with SS and successfully treated with oral glucocorticoids.

## Case presentation

**Figure 1:**
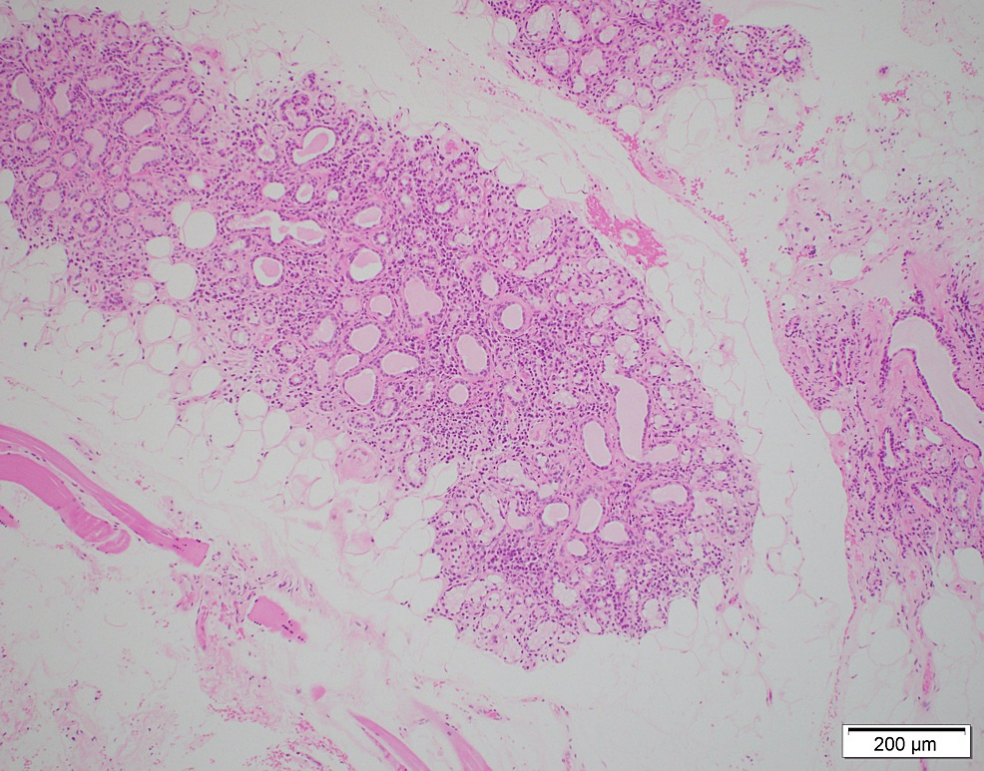
Low-power view showing salivary gland lobules with lymphocytic infiltration and partial fibrosis.

**Figure 2:**
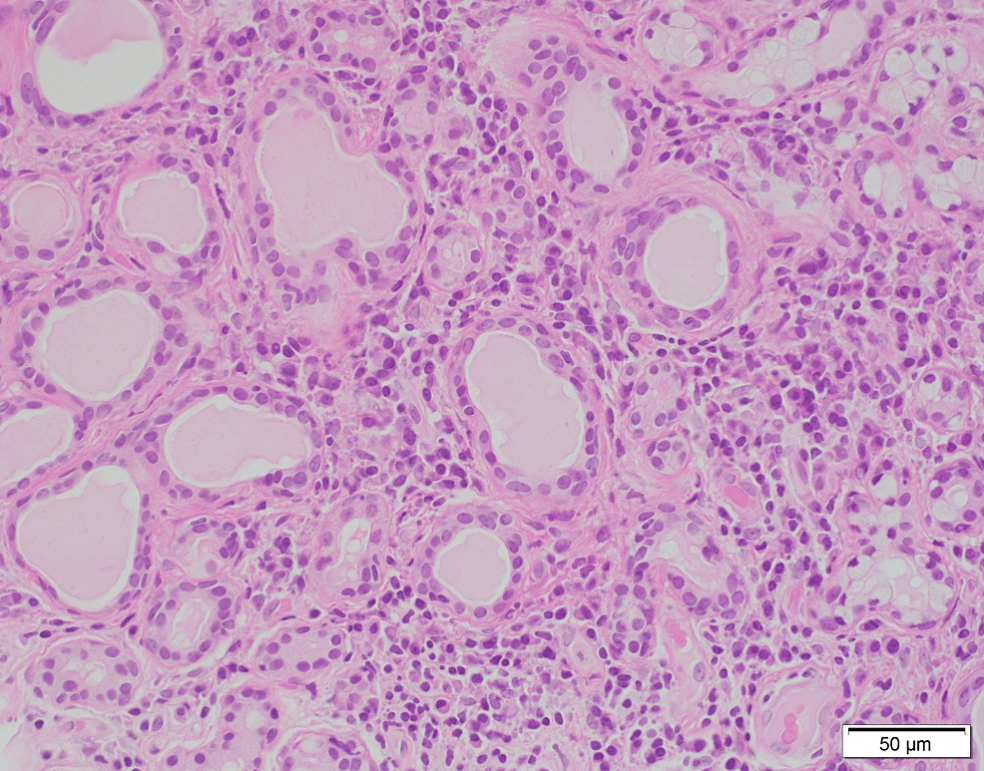
High-power view showing the ductal cells of the salivary gland characteristically surrounded by lymphocytic cells and plasma cells.

An 84-year-old male, with end-stage renal disease and in a hemodialysis program for eight years, was referred to our hospital with a four days history of fever and generalized malaise. He had been taking only nutritional supplements for anorexia that lasted over a year. He had a history of hypertension, which was treated with azilsartan 20 mg and amlodipine 7.5 mg. He was fully conscious, body temperature was 37.8 ℃, blood pressure was 120/65 mmHg, heart rate was 65 beats per minute, and respiratory rate was 16 breaths per minute. Physical examination revealed no abnormalities. Chest X-ray and whole-body computed tomography were unremarkable. His serum C-reactive protein level and procalcitonin level was elevated (4.07 mg/dl, normal 0-0.3; and 0.3 ng/ml, normal 0-0.25), serum immunoglobulin (Ig) G and A were elevated (30.29 g/L, normal 8.6-17.4; and 6.15 g/L, normal 0.93-3.93), and Ig M was declined (0.42 g/L, normal 0.5-2.7) (Table 1). Blood culture and multiple nasal swab polymerase chain reaction (PCR) tests for coronavirus disease 2019 (COVID-19) were all negative. We diagnosed him with fever without source on admission and started treatment with ceftriaxone and azithromycin as empirical therapy. The results of the other laboratory tests taken on day zero were known on day three. Blood immunochemistry showed that anti-nuclear antibody (1:2560, homogenous and specked) and anti-SS-A and B antibodies (>240 U/ml and >320 U/ml) were positive. The Schirmer test was positive, indicating decreased lacrimal fluid. A lip biopsy showed lymphocyte and plasma cellular infiltrations in the minor salivary glands, and his saliva volume had decreased. His focus score, which is the number of lymphocytic foci per 4 mm^2^, was two (Figures 1, 2).

**Table 1:** Laboratory investigations for the patient. ND: not done; Anti-SS-B: anti-Sjögren's syndrome type B; scl: scleroderma

Variable	Reference Range	On admission	Hospital Day 3	Hospital Day 28	Hospital Day 38	Hospital Day 56
Hemogrobin (g/dl)	11.6-14.8	10.3	9	9.1	8.3	9
Hematocrit (%)	35.1-44.4	31	26.7	27.7	25	27.8
White blood cell count (per μl)	3000-8600	5900	6200	8400	9400	8500
Neutrophil count (per μl)	1800-7500	4425	4433	6989	7952	7148
Lymphocyte count (per μl)	1000-4800	708	955	823	1090	969
Platelet count (per μl)	158,000-348,000	236,000	198,000	157,000	216,000	141,000
Creatinine (mg/dl)	0.46-0.79	3.53	5.54	4.6	4.9	3.8
Urea nitrogen (mg/dl)	8-22	23.8	43.4	43.2	44.5	42.5
Albmin (g/dl)	4-5	2.5	2.3	2	1.6	1.9
C-reactive protein (mg/dl)	0-0.3	4.07	3.5	12.7	5.66	0.62
IgG (mg/dl)	700-1600	3029	ND	ND	ND	2113
IgA (mg/dl)	100-490	615	ND	ND	ND	560
IgM (mg/dl)	50-320	42	ND	ND	ND	45
C3 (mg/dl)	50-120	98	ND	ND	ND	105
C4 (mg/dl)	13-54	27	ND	ND	ND	31
Antinuclear antibody	Negative	1:2560 (homogenous and speckled pattern)	ND	ND	ND	ND
Anti-double stranded DNA antibody	Negative	ND	Negative	ND	ND	ND
Anti-SS-A (Ro) antibody (U/ml)	Negative	ND	>240	ND	ND	ND
Anti-SS-B (La) antibody (U/ml)	Negative	ND	>320	ND	ND	ND
Anti-scl-70 antibody	Negative	ND	Negative	ND	ND	ND
Anti-jo-1 antibody	Negative	ND	Negative	ND	ND	ND

The diagnosis of SS was confirmed on day 13. However, he was infected with COVID-19 on day 11, and treatment with remdesivir for three days was commenced. He had aspiration pneumonia on day 28, so he was treated with ampicillin sodium and sulbactam sodium for 10 days. Based on the European League Against Rheumatism (EULAR) SS Disease Activity Index (ESSDAI) score of 8, the patient was determined to have moderate systemic involvement, and treatment with oral glucocorticoids 10 mg was initiated on day 42. He could only take nutritional supplements of 300-500 kcal/day the day before but was able to consume an 800-kcal meal after treatment started, and his expression brightened on day 44. All symptoms resolved and he was discharged on day 60. We tapered the systemic glucocorticoids to 5 mg/day over two months and then maintained. He is under regular follow-up without relapse.

## Discussion

In this report, we describe a male patient referred with anorexia, fever, and generalized malaise who was diagnosed with SS. The overall prevalence rate of primary SS is about 60 cases per 100,000 and it is therefore a common connective tissue disease [5]. Nevertheless, it can be difficult to diagnose SS as the symptoms are frequently unspecific and diagnostic markers are lacking in many patients [6]. In this case, saliva and lachrymal volume were markedly decreased and there must have been dryness symptoms, but he was unaware of them. It is possible that he was less likely to notice the dryness of his mouth and eyes because he was elderly and a dialysis patient.

The 2016 American College of Rheumatology/EULAR classification criteria for primary SS is used for diagnosis [7]. Salivary gland biopsy is useful for the diagnosis of SS with a sensitivity of 87.4% and specificity of 87.3% [8]. Although not included in the diagnostic criteria, B-cell dysfunction and hyperactivity are also distinctive features of SS. Hypergammaglobulinemia, hypocomplementemia, and raised levels of kappa-free light chains and beta 2 microglobulin indicate B-cell hyperactivity [9]. In this case, elevated levels of gamma globulin and beta-2 microglobulin and characteristic pathologic findings were observed (Figures 1, 2). In particular, the presence of both fibrosis and lymphocytic infiltration revealed a combination of chronic and acute lesions. We thought it reflected a history of anorexia for a year, and fever and general malaise for several days.

Limited controlled therapeutic trials have been conducted for SS, so the optimal treatment is not yet clear [6]. EULAR recommends the use of systemic therapies such as glucocorticoids, antimalarials, immunosuppressive agents, intravenous immunoglobulins, and biologics for patients with active systemic disease [10]. Since only glucocorticoids are approved for the treatment of SS in Japan, we could not use the other treatments. The ESSDAI is now the gold standard for measuring disease activity and assessing outcomes in clinical research [11]. The patient had an ESSDAI score of 8 points, indicating moderate disease activity, and he was treated with systemic glucocorticoids. He had not been able to eat a normal diet for a year, but he had not been treated because it was considered to be due to his age and his being on dialysis. Treatment of oral glucocorticoids allowed him to consume a normal diet, and he was very pleased with the therapeutic effect. Diagnosis of SS is sometimes difficult, but clinicians should consider the possibility of SS when seeing the above non-specific symptoms.

## Conclusions

SS can be difficult to diagnose due to its varied symptoms, which include fatigue, diffuse pain, fever, and shortness of breath. Elderly people and dialysis patients in particular are less likely to be aware of sicca symptoms and may not have been diagnosed. Undiagnosed SS patients may experience significant symptom improvement with proper treatment. Therefore, SS should be considered in the differential diagnosis of these non-specific symptoms.

## References

[1] Ng KP, Isenberg DA (2008). Sjögren's syndrome: diagnosis and therapeutic challenges in the elderly. Drugs Aging.

[2] François H, Mariette X (2016). Renal involvement in primary Sjögren syndrome. Nat Rev Nephrol.

[3] Cheng MH, Lin JH, Yen TH (2014). Thrombotic microangiopathy complicating newly diagnosed Sjögren's syndrome in a dialysis patient. Ren Fail.

[4] Kinoshita Y, Ikeda T, Miyamura T (2022). Nodular pulmonary amyloidosis associated with Sjögren's syndrome. Intern Med.

[5] Qin B, Wang J, Yang Z, Yang M, Ma N, Huang F, Zhong R (2015). Epidemiology of primary Sjögren's syndrome: a systematic review and meta-analysis. Ann Rheum Dis.

[6] Witte T (2019). Sjögren's syndrome (Article in German). Z Rheumatol.

[7] Shiboski CH, Shiboski SC, Seror R (2017). 2016 American College of Rheumatology/European League Against Rheumatism classification criteria for primary Sjögren's syndrome: a consensus and data-driven methodology involving three international patient cohorts. Arthritis Rheumatol.

[8] Fujibayashi T, Sugai S, Miyasaka N, Hayashi Y, Tsubota K (2004). Revised Japanese criteria for Sjögren's syndrome (1999): availability and validity. Mod Rheumatol.

[9] Kroese FG, Abdulahad WH, Haacke E, Bos NA, Vissink A, Bootsma H (2014). B-cell hyperactivity in primary Sjögren's syndrome. Expert Rev Clin Immunol.

[10] Ramos-Casals M, Brito-Zerón P, Bombardieri S (2020). EULAR recommendations for the management of Sjögren's syndrome with topical and systemic therapies. Ann Rheum Dis.

[11] Seror R, Ravaud P, Mariette X (2011). EULAR Sjogren's syndrome patient reported index (ESSPRI): development of a consensus patient index for primary Sjogren's syndrome. Ann Rheum Dis.

